# Modifiable risk factors for epilepsy: A two‐sample Mendelian randomization study

**DOI:** 10.1002/brb3.2098

**Published:** 2021-03-02

**Authors:** Shuai Yuan, Torbjörn Tomson, Susanna C. Larsson

**Affiliations:** ^1^ Unit of Cardiovascular and Nutritional Epidemiology Institute of Environmental Medicine Karolinska Institutet Stockholm Sweden; ^2^ Department of Clinical Neuroscience Karolinska Institutet Stockholm Sweden; ^3^ Unit of Medical Epidemiology Department of Surgical Sciences Uppsala University Uppsala Sweden

**Keywords:** depression, epilepsy, iron, Mendelian randomization analysis, smoking

## Abstract

**Introduction:**

We conducted a two‐sample Mendelian randomization study to determine the associations of modifiable risk factors with epilepsy.

**Methods:**

Fourteen potential risk factors for epilepsy were selected based on a systematic review of risk factors for epilepsy. Single‐nucleotide polymorphisms associated with each exposure at the genome‐wide significance threshold (*p* < 5×10^–8^) were proposed as instrumental variables from corresponding genome‐wide association studies. Summary‐level data for epilepsy were obtained from the FinnGen consortium (4,588 cases and 144 780 noncases). Potential causal associations (*p* < .05) were attempted for replication using UK Biobank data (901 cases and 395 209 controls).

**Results:**

Among 14 potential risk factors, 4 showed significant associations with epilepsy in FinnGen. All associations were directionally similar in UK Biobank and associated with epilepsy at *p* ≤ .004 in meta‐analyses of FinnGen and UK Biobank data. The odds ratios of epilepsy were 1.46 (95% CI, 1.18, 1.82) for one unit increase in log odds ratio of having depression, 1.44 (95% CI, 1.13, 1.85) for one standard deviation increase in serum ferritin, 1.12 (95% CI, 1.04, 1.21) for one standard deviation increase in transferrin saturation, and 1.25 (95% CI, 1.09, 1.43) for one standard deviation increase in the prevalence of smoking initiation. There were suggestive associations of serum iron and magnesium with epilepsy. No association was observed for insomnia, blood pressure, alcohol consumption, or serum vitamin B12, 25‐hydroxyvitamin D and calcium levels.

**Conclusion:**

This MR study identified several modifiable risk factors for adulthood epilepsy. Reducing prevalence of depression and smoking initiation should be considered as primary prevention strategies for epilepsy.

## Graphical abstract

Genetic predisposition to depression and smoking initiation were associated with an increased risk of epilepsy. Genetic predicted higher iron status was associated with an increased risk of epilepsy. There was a suggestive association of serum magnesium with epilepsy.

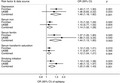

## 1 INTRODUCTION

Epilepsy is one of the most common serious central nervous system diseases affecting all ages (Thijs et al., 2019). It is estimated that epilepsy has an incidence around 50 per 100,000 per year across different regions affecting over 70 million people worldwide in 2019 (Thijs et al., 2019). Despite the availability of more than 25 different antiseizure medications (ASMs), more than 30% of people with epilepsy under treatment continue to have seizures (E. Perucca et al., 2020) and many of those rendered seizure‐free suffer from adverse drug effects (P. Perucca & Gilliam, 2012). In addition, approximately 80% of epilepsy patients live in low‐income and middle‐income countries where treatment in many cases is unavailable (Saxena & Li, 2017). Therefore, it is of great importance to pinpoint modifiable risk factors for epilepsy from the perspective of primary prevention.

Stroke, head trauma, cerebral infection, and brain tumors have been identified as causes of epilepsy at any age, but the etiology remains unknown in about 50% of newly diagnosed epilepsy cases (Duncan et al., 2006). Epidemiological data on other risk factors, especially modifiable risk factors for adult epilepsy, are scarce and conflicting (Duncan et al., 2006; Subota et al., 2017). In addition, findings of observational studies are prone to be biased by residual confounding and reverse causality. Thus, whether established associations are causal often remains unclear and needs verification.

Utilizing genetic variants as instrumental variables, the Mendelian randomization (MR) design can distinguish correlation from causation in observational data by diminishing residual confounding and reducing reserve causality (Davey Smith & Ebrahim, 2005). The finding of an MR analysis is less likely to be biased by confounding because genetic variants are randomly allocated at conception and, therefore, one trait is generally not correlated to other traits. This process resembles the random assignment of participants to experimental and control groups in a randomized controlled trial (Figure S1; Davey Smith & Ebrahim, 2005; Stephen Burgess, 2015). Individuals with a genetic variation that leads to a higher level of the risk factor (e.g., higher serum iron) will on average be exposed to a higher level of the risk factor compared with those with the genetic variation related to lower level of the risk factor. An MR analysis also avoids reverse causality as alleles are fixed and cannot be modified by the onset or progression of the disease (Davey Smith & Ebrahim, 2005; Stephen Burgess, 2015). Hence, if a genetic variant that alters the level of the exposure or imitates its biological effects is also related to the disease, this offers strong evidence that the exposure is a cause of the disease. Here, we conducted an MR study to determine the causal associations of several modifiable risk factors with risk of epilepsy.

## 2 METHODS

### 2.1 Study design

We firstly conducted a systematic review of studies on risk factors for epilepsy published in recent 5 years in the PubMed database to pinpoint possible epilepsy‐related risk factors. One hundred out of 1849 articles were included and provided information of 84 possible risk factors for epilepsy. Detailed information on the systematic review is provided in supplementary method. Fourteen of the 84 possible risk factors were selected by two criteria: 1) a potentially modifiable risk factor, and 2) with more than 3 instrumental variables. We then performed MR analyses to investigate the associations of 14 modifiable factors with risk of epilepsy using FinnGen consortium data (the discovery stage). For traits associated with epilepsy, we attempted to replicate their associations with epilepsy using data from UK Biobank (the replication stage). This MR study is based on summary‐level (i.e., aggregated) data only and has been approved by the Swedish Ethical Review Authority.

### 2.2 Outcome sources

Summary‐level data for epilepsy in the discovery stage were obtained from R3 release of FinnGen consortium with 4,588 epilepsy cases and 144 780 noncases of Finnish ancestry (consortium, 2020). The diagnosis of epilepsy in FinnGen was defined by G40 in International Classification of Diseases (ICD) 10th version and the genotype data was obtained from Finnish biobanks and digital health record data from Finnish health registries. Association tests were adjusted for age, sex, ten genetic principal components, and genotyping batch. Data from UK Biobank (round 1) were used in the replication stage and included 901 cases of epilepsy (defined based on the ICD code G40) and 395 209 controls of European ancestry (http://pheweb.sph.umich.edu/SAIGE‐UKB/pheno/345.1).

### 2.3 Instrumental variable selection

Single‐nucleotide polymorphisms (SNPs) associated with each exposure at the genome‐wide significance threshold (*p* < 5×10^–8^) were proposed as instrumental variables for 14 exposures from corresponding genome‐wide association studies (GWASs). For SNPs in linkage disequilibrium (*r^2^* ≥ .01), only the SNP with lowest p‐value was used. For each exposure, SNPs were harmonized so that the effect alleles reflected the allele associated with an increased probability, prevalence, or levels of the exposure. Detailed information for included GWASs and instrumental variables used is shown in Table S1 and Table 1.

**TABLE 1:** Data sources of the instrumental variables

Exposure	Sample size	SNPs ^a^	Overlap (%) ^b^	Variance	*F*‐statistic ^c^	OR of 80% power ^d^
Depression	807,553	97/84	~49	NA	NA	NA
Insomnia	1,331,010	248/202	~30	2.6	16/43	NA
Systolic blood pressure	955,229	244/214	~41	>4.0	25/68	NA
Diastolic blood pressure	955,162	300/258	~41	>4.0	21/55	NA
Serum vitamin B12	45,576	15/12	0	6.3	669/1775	<0.84 or > 1.17
Serum 25‐hydroxyvitamin D	79,366	7/7	0	5.3	1194/3167	<0.82 or > 1.18
Serum 25‐hydroxyvitamin D	42,274					
Serum iron	48,972	5/5	0	3.4	1051/2788	<0.77 or > 1.23
Serum ferritin	48,972	6/6	0	0.9	226/600	<0.56 or > 1.45
Serum transferrin saturation	48,972	5/5	0	6.9	2214/5871	<0.84 or > 1.16
Serum transferrin	48,972	8/8	0	7.2	1449/3841	<0.84 or > 1.16
Serum calcium	15,366	6/6	0	1.6	405/1073	<0.67 or > 1.34
Serum magnesium	39,400	7/7	0	0.9	194/514	<0.56 or > 1.45
Alcohol consumption	941,280	99/79	~42	2.5	39/103	<0.74 or > 1.27
Smoking initiation	1,232,091	378/295	~32	4.0	16/44	<0.79 or > 1.21

Abbreviations: NA, not available; SNPs, single‐nucleotide polymorphisms.

^a^ Number of SNPs identified in GWAS / Number of SNPs used in the present Mendelian randomization study.

^b^ Overlap with UK Biobank (there was no sample overlap between GWASs on exposure and FinnGen).

^c^ F‐statistic in FinnGen / *F*‐statistic in UK Biobank (the calculation was based on all identified SNPs and assuming identical variance explained by these SNPs in GWASs on exposure and in outcome data).

^d^ OR of 80% power in FinnGen (the discovery stage) and the power was estimated using the weltool: https://shiny.cnsgenomics.com/mRnd/. Power estimation was inapplicable for depression, insomnia, and blood pressures since the unit of effect sizes of instrumental variables for these exposures were not in standard deviation.

### 2.4 Statistical analysis

The inverse‐variance weighted method with random effects was used as the main analysis (Burgess et al., 2017). As inverse‐variance weighted method is sensitive to invalid instrumental variables and pleiotropic bias, two sensitivity analyses, weighted median and MR‐Egger approaches, were performed (Bowden et al., 2016; Burgess & Thompson, 2017). Weighted median method provides an accurate estimate of the causal effect of the exposure on outcome when more than 50% of the weight in the analysis comes from valid instrumental variables (Bowden et al., 2016). MR‐Egger regression can detect and correct for directional pleiotropy; however, the estimation is of low precision (Burgess & Thompson, 2017). The Cochrane *Q* statistic was used to explore heterogeneity among SNPs for each trait, and horizontal pleiotropy was defined according to the *p*‐value for the intercept in MR‐Egger model (Burgess et al., 2017). The *F*‐statistic and power were estimated (Table 1; Brion et al., 2013; Burgess et al., 2016). The odds ratios (ORs) and their 95% confidence intervals (CIs) were scaled to one unit increase for each trait listed in Table S1. The associations with *p* < .05 in the main analysis in FinnGen were taken forward to the replication stage using data from UK Biobank. For those associations, we performed meta‐analyses of FinnGen and UK Biobank data. All analyses were performed in Stata/SE 15.0 using the mrrobust package (Spiller et al., 2019). All estimates were reported with *p*‐values in two‐tailed tests. We considered associations with *p* ‐values below 0.004 (where *p* = .05/14 risk factors) to represent strong evidence of causal associations, and associations with *p* values below .05 but above .004 as suggestive evidence of associations. We also interpreted the results based on a combinational consideration of the statistical significance, the consistency across sensitivity analyses, the agreement between findings in FinnGen and UK Biobank, and the significance of the results in the meta‐analyses of the two data sources.

## 3 RESULTS

### 3.1 Discovery stage

Among 14 possible modifiable risk factors, 5 factors were significantly associated with risk of epilepsy in the discovery dataset (FinnGen consortium). Results from the main (inverse‐variance weighted) and sensitivity analyses are presented in Table 2. Specifically, genetically predisposition to depression and smoking initiation, and high levels of serum iron, ferritin, and transferrin saturation were associated with increased risk of epilepsy. For one unit increase in log OR of depression, the OR of epilepsy was 1.49 (95% CI, 1.17, 1.90) in the inverse‐variance weighted model. The ORs of epilepsy for one standard deviation increases of the iron status biomarkers were 1.15 (95% CI, 1.02, 1.29) for serum iron, 1.37 (95% CI, 1.05, 1.80) for serum ferritin, and 1.12 (95% CI, 1.03, 1.21) for serum transferrin saturation. The OR was 1.19 (95% CI, 1.02, 1.38) for one standard deviation increase in the prevalence of smoking initiation. There was a suggestive association between higher serum magnesium and risk of epilepsy in the weighted median model, with an OR of 0.56 (95% CI, 0.36, 0.86) for one standard deviation increase of serum magnesium levels. The associations remained consistent in all analyses (Table 2). We detected moderate heterogeneity in the analysis of insomnia and diastolic blood pressure, and possible pleiotropy in the MR‐Egger analysis of depression (Table S2). After correction for pleiotropy, the association between genetic liability to depression and risk of epilepsy became stronger (Table 2). There was limited evidence supporting associations of other modifiable factors with epilepsy.

**TABLE 2:** Associations of 14 modifiable factors with epilepsy in Mendelian randomization analyses (discovery stage based on FinnGen data)

Exposure	IVW method			Weighted median method			MR‐Egger regression		
	OR	95% CI	*p*	OR	95% CI	*p*	OR	95% CI	*p*
Depression	1.49	1.17–1.90	.001	1.49	1.05–2.11	.027	4.42	1.49–13.11	.009
Insomnia	1.07	0.98–1.17	.115	1.13	0.99–1.28	.066	1.22	0.87–1.73	.255
Systolic blood pressure	1.13	0.97–1.33	.121	1.05	0.83–1.34	.677	1.78	0.96–3.28	.068
Diastolic blood pressure	1.01	0.77–1.31	.968	1.12	0.76–1.63	.573	1.48	0.61–3.58	.388
Serum vitamin B12	1.00	0.84–1.18	.998	1.04	0.87–1.23	.693	0.98	0.67–1.44	.925
Serum 25‐hydroxyvitamin D	1.01	0.84–1.21	.902	0.98	0.82–1.16	.797	0.96	0.69–1.35	.843
Serum iron	1.15	1.02–1.29	.022	1.12	0.93–1.35	.250	1.34	0.97–1.84	.177
Serum ferritin	1.37	1.05–1.80	.020	1.57	1.08–2.28	.019	2.06	1.03–4.12	.109
Serum transferrin saturation	1.12	1.03–1.21	.010	1.12	0.97–1.30	.122	1.25	1.01–1.53	.129
Serum transferrin	0.95	0.87–1.04	.274	1.00	0.90–1.12	.936	1.00	0.87–1.15	.980
Serum calcium	0.93	0.70–1.24	.620	1.02	0.68–1.52	.936	1.02	0.55–1.90	0.951
Serum magnesium	0.78	0.51–1.20	.258	0.64	0.44–0.94	.023	0.87	0.22–3.49	.857
Alcohol consumption	1.54	0.99–2.41	.058	1.53	0.76–3.08	.236	3.00	0.96–9.32	.061
Smoking initiation	1.19	1.02–1.38	.023	1.24	1.00–1.55	.055	1.39	0.74–2.62	.302

Abbreviations: CI, confidence interval; IVW, inverse‐variance weighted; OR, odds ratio.

### 3.2 Replication stage and meta‐analyses

**FIGURE 1:**
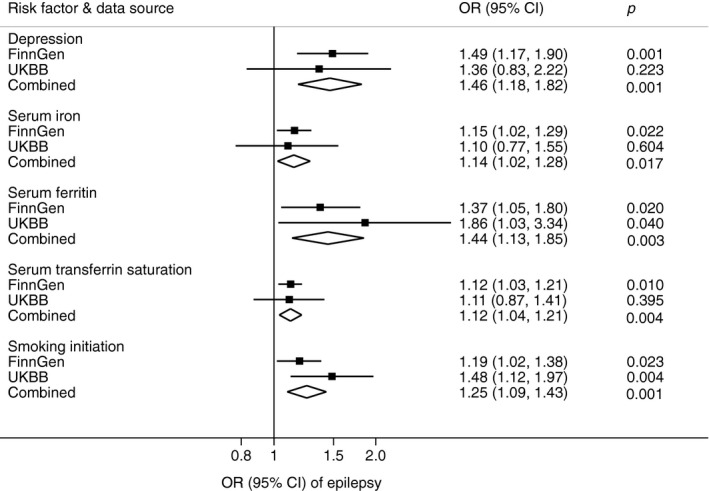
Combined odds ratios of epilepsy for seven modifiable factors from both FinnGen consortium and UK Biobank. CI indicates confidence interval; OR, odds ratio; UKBB, UK Biobank

Of the 5 potential risk factors for epilepsy identified in the discovery stage, all were replicated (same direction and of broadly similar magnitude of association) (Table S3). The combined associations of genetic liability to depression and smoking initiation and genetically predicted higher levels of serum ferritin and transferrin saturation with epilepsy persisted at *p* ≤ .004 in meta‐analyses of data from the two sources (Figure 1). The combined ORs of epilepsy were 1.46 (95% CI, 1.18, 1.82) for one unit increase in log OR of having depression, 1.44 (95% CI, 1.13, 1.85) for one standard deviation increase in serum ferritin, 1.12 (95% CI, 1.04, 1.21) for one standard deviation increase in transferrin saturation, and 1.25 (95% CI, 1.09, 1.43) for one standard deviation increase in the prevalence of smoking initiation.

## 4 DISCUSSION

In the present MR study, we identified 4 out of 14 possible causal risk factors for epilepsy. In detail, genetic liability to depression and smoking initiation and genetically predicted serum ferritin and transferrin saturation showed robust positive associations with epilepsy risk. There were suggestive associations of serum iron and magnesium with epilepsy. This is the first MR study that comprehensively explores the role of possible modifiable risk factors for epilepsy.

A high prevalence of depression among patients with epilepsy has been revealed (Robertson & Trimble, 1983). Recent studies have further verified a positive association between depression and epilepsy, albeit with limited evidence supporting the causality due to unobserved confounding (Hesdorffer et al., 2000; Josephson et al., 2017). The present study using MR analysis confirmed the robust causal association between them. In addition, studies stated that the rate of genetic predisposition to depression is higher in families with epilepsy patients compared to families without epilepsy history (Kanner, 2003). Even though the development of epilepsy mostly relies on the interaction between genetic and environmental factors (Kanner, 2003), paying more attention to depression prevention in individuals with family history of epilepsy may be a useful strategy to reduce related epilepsy.

Iron status has been scarcely studied as an etiological risk factor for epilepsy or for provoked seizure. A case‐control study including 75 children with first febrile seizure and 75 controls found a possible role of iron deficiency in first febrile seizure (Daoud et al., 2002). Nonetheless, our MR findings showed a robust causal association between high iron status (serum ferritin and transferrin saturation) and increased risk of epilepsy. High serum iron levels have been found to be causally associated with an increased risk of stroke (Gill et al., 2018), which, in turn, may increase the risk of epilepsy. Another explanation could be a detrimental effect of inflammation caused by high‐iron status on epilepsy. Increased iron stores are correlated with markers of chronic inflammation (Wessling‐Resnick, 2010), which plays a vital role in epilepsy (Vezzani et al., 2011).

With regard to magnesium, a cohort study of 2,442 middle‐aged Finnish men followed up for an average of 22.4 years showed an inverse association between dietary magnesium intake and risk of developing epilepsy (Yary & Kauhanen, 2019). Our MR evidence confirmed an inverse association between genetically predicted serum magnesium and epilepsy in the FinnGen consortium, but the association was not replicated in UK Biobank. Thus, whether magnesium plays a causal role in preventing epilepsy needs further study.

Smoking has been associated with epilepsy in observational studies. A cross‐sectional study including 429 adults with epilepsy found that the prevalence of current smoking was ~ 13% higher in epilepsy patients compared to the general population with the prevalence of ~ 19% (Torriani et al., 2016). A cohort study with 116 363 women found a positive association between current smoking and risk of seizure, and between past smoking and epilepsy after adjustment for stroke and other potential confounders (Dworetzky et al., 2010). Midlife smoking was associated with a 9% higher risk of late‐onset epilepsy in a recent prospective study comprising 10,420 adults (Johnson et al., 2018). The present MR study provides evidence that the association between smoking and epilepsy is causal. A possible explanation for this positive association is a direct effect of nicotine on glutamate release. Animal experimental data have shown that a low dose of nicotine delayed the onset of seizures (de Fiebre & Collins, 1988), whereas, at high doses, nicotine caused convulsions (Broide et al., 2002). A review also noted that the mechanism underlying the association between smoking and epilepsy is complicated and deserves more investigation (Rong et al., 2014).

There are three main assumptions for MR studies: (1) the genetic variants utilized as instrumental variables should be robustly associated with the risk factor of interest (Relevance assumption); (2) the selected genetic variants should not be associated with potential confounders (Independent assumption); and (3) the genetic variants should affect the risk of the outcome only through the risk factor, not via alternative pathways (Exclusion restriction assumption) (Figure S1). With regard to the exclusion restriction assumption, we cannot rule out that pleiotropy biased our findings although results from sensitivity analyses remained consistent and no pleiotropic effect was not observed by the MR‐Egger model, except for the analysis of depression in FinnGen.

There are several strengths of this study. This is the first study using MR methodology that has comprehensively assessed the modifiable associations for epilepsy and most potential causal associations were replicated in an independent population. Additionally, we performed several sensitivity analyses to test the consistency of results and correct for possible pleiotropy.

This study has limitations. We confined the time of literature search on risk factors for epilepsy to the previous 5 years, and, therefore, cannot rule out that we might have missed some risk factors for epilepsy that were not studied in recent years. In addition, we confined the literature search topic using “risk factor,” and possibly missed studies that did not specify “risk factor” in the title or abstract. The major limitation is that we might have overlooked weak associations, especially for exposures with small variance explained by used SNPs. The replication study included a limited number of epilepsy cases, which resulted in low precision of the estimates and the possibility of false‐negative error. For traits with instrumental variables selection based on a mixed population, population bias might be introduced. However, the European‐descent population made up the majority in used corresponding GWASs and most association tests adjusted for population principal components, which reduced the risk that our findings might be biased by population structures. Epilepsy diagnosis is based on ICD codes from administrative databases, and these have not been validated individually. The validity of the ICD code for epilepsy has, however, been assessed in patient registries in Sweden and Denmark and found to be high with a positive predictive value for epilepsy in the Swedish Inpatient Register (main part of the Swedish Patient Register) estimated at 79% (Nilsson et al., 1997). In Denmark, a positive predictive value for epilepsy of 81% (Christensen et al., 2007). While G40 represents all epilepsies, we acknowledge that epilepsy is a heterogeneous disease with different etiologies and presumably different risk factors. Genetic instruments and outcome datasets were derived from genome‐wide association studies including adults. Thus, our findings may not be generalizable to children.

In summary, the present MR study identified four modifiable risk factors for adulthood epilepsy. Reducing prevalence of depression and smoking initiation should be considered as primary prevention strategies for epilepsy. The positive association between iron status and epilepsy needs verification. Whether increased magnesium intake can prevent epilepsy needs further study.

## CONFLICT OF INTERESTS

All authors declare no competing interests. Dr. Tomson reports grants from Eisai, grants from GSK, grants from UCB, grants from Bial, personal fees from Eisai, personal fees from Sanofi, personal fees from Sun Pharma, personal fees from UCB, personal fees from Sandoz, grants from EU, grants from Stockholm County Council, grants from CURE, all outside the submitted work. We confirm that we have read the Journal's position on issues involved in ethical publication and affirm that this report is consistent with those guidelines.

## AUTHOR CONTRIBUTIONS

SY and SCL designed the study and contributed to the interpretation of the results and critical revision of the manuscript for important intellectual content. SY performed the statistical analyses and drafted the manuscript. TT contributed with critical review of the interpretation and the manuscript and for revising the manuscript for intellectual content. All authors and approved the final version of the manuscript.

## DATA SHARING

The datasets analyzed in this study are publicly available summary statistics.

### Peer Review

The peer review history for this article is available at https://publons.com/publon/10.1002/brb3.2098.

## Supporting information

Supplementary Material

## Data Availability

Genetic instruments can be obtained from the individual referenced papers. Summary‐level genetic data for epilepsy from the FinnGen consortium can be downloaded at https://www.finngen.fi/fi. Replication stage data from UK Biobank are available at http://pheweb.sph.umich.edu/SAIGE‐UKB/.
