# Supplementary material for: Modifiable risk factors for epilepsy: A two‐sample Mendelian randomization study
Source: Brain Behav. 2021 Mar 2;11(5):e02098. doi: 10.1002/brb3.2098 (PMC8119863; doi:10.1002/brb3.2098)

**Supplements**

**Causal effects of risk factors on epilepsy: a two-sample Mendelian randomization study**

*Shuai Yuan, Torbjörn Tomson, Susanna C. Larsson*

**Supplementary method for systematic review**

**Supplementary Table 1. Detail information of included genome-wide association studies**

**Supplementary Table 2. Heterogeneity and pleiotropy in studied associations of risk factors with epilepsy in FinnGen**

**Supplementary Table 3. Associations of risk factors with epilepsy in replication stage using UK Biobank data**

**Supplementary Figure 1. Rationale and assumptions of Mendelian randomization analysis**

**Supplementary method for systematic review**

**Search strategy**

((((Epilepsy[MeSH Terms] OR Late-onset epilepsy[Title/Abstract]) OR Seizure[MeSH Terms] OR Seizure[Title/Abstract])) AND (Risk Factors[MeSH Terms] OR Risk Factors[Title/Abstract] OR Risk Factor[Title/Abstract])) AND "last 5 years"[PDat])

In total, 1849 studies have been identified by a search in PubMed using above search strategy. After selection, 100 studies were included and 84 possible risk factors for epilepsy were pinpointed as following:

| **Health status** | **Biomarkers** | **Lifestyle** | **Other factors** |
| --- | --- | --- | --- |
| Stress | Folic acid | Alcohol use | Sex |
| Depression | Vitamin B12 | Smoking | Socioeconomic status |
| Anxiety | Vitamin C | Physical activity | Unspecified sepsis |
| Sleep problems | 25-hydroxyvitamin D |  | Family or personal history of epilepsy |
| Migraine | Iron status |  | Complex febrile seizures |
| Pain | Zinc |  | Low ambient temperature |
| Personality disorders | Copper |  | Intestinal microbiome |
| Birth weight | Magnesium |  | Brain tumor |
| Autoimmune disorders | Serum sodium |  | Atypical neuroleptics |
| Asthma | Fasting glucose |  | Anti-depressants |
| Celiac disease | Hemoglobin level |  | Surgery or trauma |
| Autism spectrum disorders | Serum uric acid |  | Maternal thyroid function |
| Dementia (Alzheimer's disease) | Leptin |  | Maternal overweight/obesity |
| Parkinson’s disease | Serum albumin levels |  | Maternal rheumatoid arthritis |
| Hippocampal volume loss | Glutamate |  | HIV infection |
| Cognitive impairment | Serum high-mobility group box-1 |  | Parasite infection (neurocysticercosis, malaria) |
| Dupuytren disease | Beta-2-adrenergic agonists |  | Presumed bacterial infection |
| Tourette syndrome | Apolipoprotein E |  | Genitourinary and gastrointestinal infections |
| Microcytic hypochromic anemia | Serum malondialdehyde |  | Neurodevelopmental impairment |
| Cirrhosis | Brain-derived neurotrophic factor |  | Preterm birth |
| Hyperbilirubinemia | Interleukin 1 |  | Brain injury at birth |
| Irritable bowel syndrome | Interleukin 6 |  |  |
| Unspecified sepsis | Interleukin 8 |  |  |
| Stroke (cerebrovascular disease) | Platelet count |  |  |
| Hypertension | Neutrophil-to-lymphocyte ratio |  |  |
| Hypoglycemia | Mean platelet volume/platelet count |  |  |
| Congenital heart disease | Serum toll-like receptor 4 |  |  |
| Diabetes (type 1 and 2) |  |  |  |
| Heart failure |  |  |  |

**Supplementary table 1. Detail information of included genome-wide association studies**

| **Exposure** | **PubMed ID** | **Year** | **Cases** | **Controls** | **Population** | **SNPs** | **Variance %** | **SD** | **Unit** |
| --- | --- | --- | --- | --- | --- | --- | --- | --- | --- |
| Depression | 30718901 | 2019 | 246363 | 561190 | European | 97 | NA | NA | Events |
| Insomnia | 30804565 | 2019 | 397972 | 933038 | European | 248 | 2.6 | NA | Events |
| Systolic blood pressure | 30224653 | 2018 | 955229 | NA | Mix | 244 | >4.0 | NA | 10 mmHg |
| Diastolic blood pressure | 30224653 | 2018 | 955162 | NA | Mix | 300 | >4.0 | NA | 10 mmHg |
| Vitamin B12 | 23754956 | 2013 | 45576 | NA | Mixed | 15 | 6.3 | NA | SD |
| 25-hydroxyvitamin D | 29343764 | 2018 | 79366 | NA | European | 6 | 2.8 | 0.33 ln-nmol/L | SD |
| 25-hydroxyvitamin D | 30526863 | 2018 | 42274 | NA | European | 1 | 2.5 | 0.33 ln-nmol/L | SD |
| Iron | 25352340 | 2014 | 48972 | NA | European | 5 | 3.4 | NA | SD |
| Ferritin | 25352340 | 2014 | 48972 | NA | European | 6 | 0.9 | NA | SD |
| Transferrin saturation | 25352340 | 2014 | 48972 | NA | European | 5 | 6.9 | NA | SD |
| Transferrin | 25352340 | 2014 | 48972 | NA | European | 8 | 7.2 | NA | SD |
| Magnesium | 20700443 | 2010 | 15366 | NA | European | 6 | 1.62 | 0.1mmoll/L | SD |
| Calcium | 24068962 | 2013 | 39400 | NA | European | 7 | 0.9 | 0.5 mmol/L | SD |
| Alcohol use | 30643251 | 2019 | 941280 | NA | European | 99 | 2.5 | NA | drinks/week |
| Smoking initiation | 30643251 | 2019 | 1232091 | NA | European | 378 | 4 | NA | Events |

NA indicates not available or not applicable; PubMed ID, PubMed identifier; SD, standard deviation; SNPs, single nucleotide polymorphisms.

**Supplementary table 2. Heterogeneity and pleiotropy in studied associations of risk factors with epilepsy in FinnGen**

| **Exposure** | **Heterogeneity** | | **Pleiotropy** | | |
| --- | --- | --- | --- | --- | --- |
|  | **Cochrane Q** | ***p*** | **Intercept** | **SE** | ***p*** |
| Depression | 89.72 | 0.288 | -0.024 | 0.012 | 0.048 |
| Insomnia | 249.28 | 0.012 | -0.006 | 0.008 | 0.440 |
| Systolic blood pressure | 205.84 | 0.625 | -0.010 | 0.007 | 0.139 |
| Diastolic blood pressure | 299.84 | 0.034 | -0.005 | 0.006 | 0.373 |
| Vitamin B12 | 17.73 | 0.088 | 0.003 | 0.025 | 0.917 |
| 25-hydroxyvitamin D | 9.88 | 0.130 | 0.008 | 0.023 | 0.748 |
| Iron | 2.03 | 0.730 | -0.025 | 0.023 | 0.359 |
| Ferritin | 3.68 | 0.597 | -0.032 | 0.025 | 0.266 |
| Transferrin saturation | 1.76 | 0.780 | -0.026 | 0.020 | 0.282 |
| Transferrin | 5.50 | 0.600 | -0.013 | 0.012 | 0.314 |
| Calcium | 4.44 | 0.617 | -0.006 | 0.018 | 0.740 |
| Magnesium | 8.47 | 0.076 | -0.008 | 0.049 | 0.881 |
| Alcohol use | 73.23 | 0.632 | -0.009 | 0.007 | 0.212 |
| Smoking initiation | 318.56 | 0.156 | -0.003 | 0.006 | 0.608 |

CI indicates confidence interval; SE, standard error.

**Supplementary table 3. Associations of risk factors with epilepsy in replication stage using UK Biobank data (901 cases and 395 209 controls)**

| **Risk factor** | **IVW-random effects** | | | **Weighted median** | | | **MR-Egger** | | | **Heterogeneity** | | **Pleiotropy** | | |
| --- | --- | --- | --- | --- | --- | --- | --- | --- | --- | --- | --- | --- | --- | --- |
|  | **OR** | **95% CI** | ***p*** | **OR** | **95% CI** | ***p*** | **OR** | **95% CI** | ***p*** | **I^2^** | **95% CI** | **Intercept** | **SE** | ***p*** |
| Depression | 1.36 | 0.83, 2.22 | 0.223 | 1.31 | 0.64, 2.66 | 0.458 | 0.78 | 0.09, 7.06 | 0.824 | 0 | 0, 26 | 0.012 | 0.025 | 0.612 |
| Iron | 1.10 | 0.77, 1.55 | 0.604 | 1.05 | 0.69, 1.6 | 0.827 | 1.55 | 0.82, 2.93 | 0.182 | 21 | 0, 66 | -0.063 | 0.051 | 0.220 |
| Ferritin | 1.86 | 1.03, 3.34 | 0.040 | 2.20 | 1.07, 4.5 | 0.031 | 2.48 | 0.79, 7.73 | 0.119 | 0 | 0, 75 | -0.027 | 0.046 | 0.562 |
| Transferrin saturation | 1.11 | 0.87, 1.41 | 0.395 | 1.10 | 0.85, 1.42 | 0.476 | 1.45 | 1.01, 2.06 | 0.041 | 12 | 0, 82 | -0.075 | 0.040 | 0.059 |
| Smoking initiation | 1.48 | 1.12, 1.97 | 0.006 | 1.77 | 1.16, 2.71 | 0.008 | 1.38 | 0.43, 4.46 | 0.589 | 0 | 0, 13 | 0.001 | 0.011 | 0.901 |

CI indicates confidence interval; IVW, inverse-variance weighted; OR, odds ratio.

We did not report MR-Egger estimates here since the power was inadequate based on the sample size in the replication stage.

**Supplementary figure 1. Rationale and assumptions of Mendelian randomization analysis**


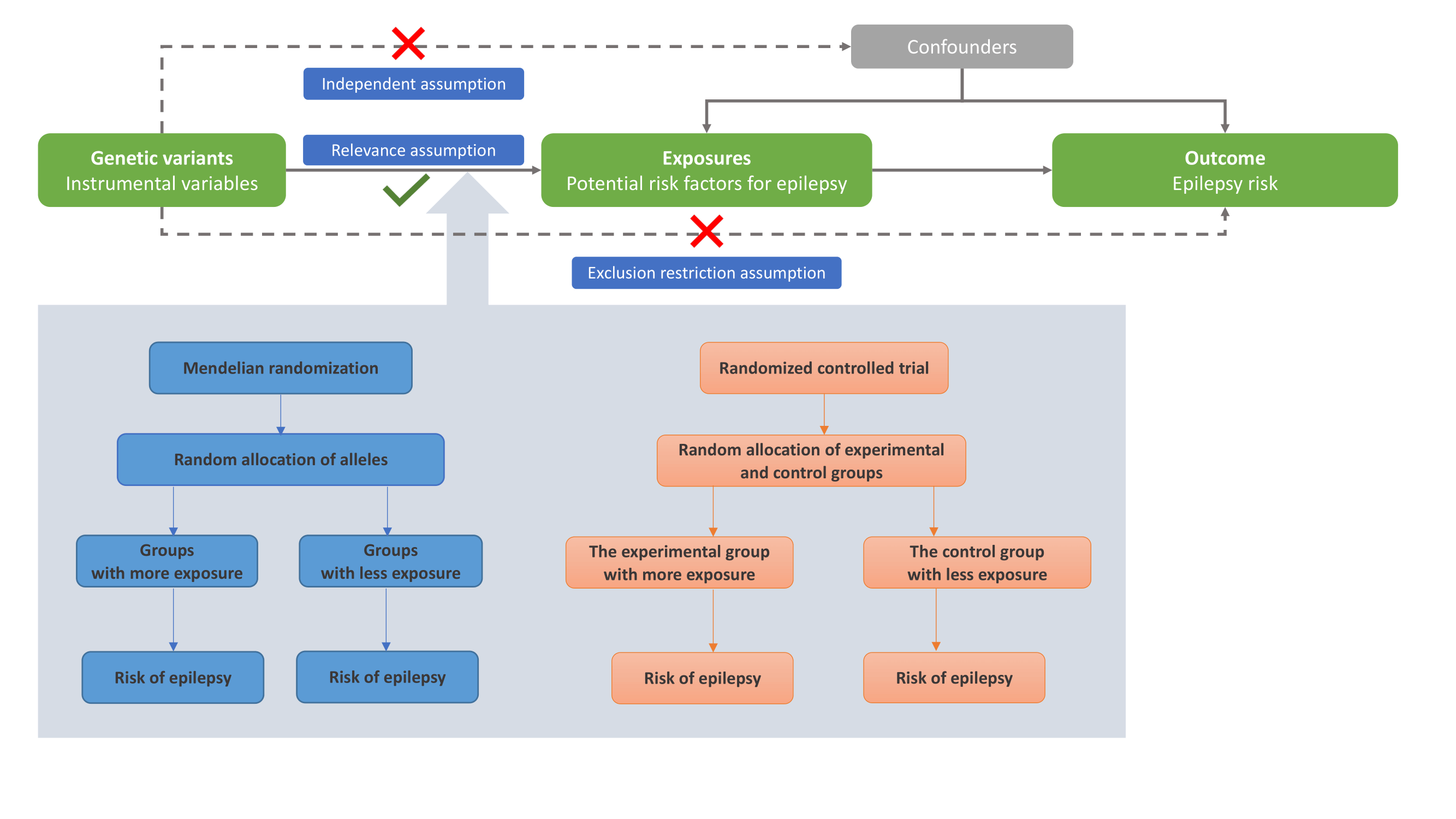

Supplement: Supplementary file 1 — Supplementary Material [file BRB3-11-e02098-s001.docx]
